# UV irradiation/cold shock-mediated apoptosis is switched to bubbling cell death at low temperatures

**DOI:** 10.18632/oncotarget.3153

**Published:** 2015-02-10

**Authors:** Szu-Jung Chen, Pei-Wen Lin, Hsin-Ping Lin, Shenq-Shyang Huang, Feng-Jie Lai, Hamm-Ming Sheu, Li-Jin Hsu, Nan-Shan Chang

**Affiliations:** ^1^ Institute of Molecular Medicine, National Cheng Kung University College of Medicine, Tainan, Taiwan, ROC; ^2^ Department of Dermatology, Chi-Mei Medical Center, Tainan, Taiwan, ROC; ^3^ Department of Dermatology, National Cheng Kung University College of Medicine, Tainan, Taiwan, ROC; ^4^ Department of Medical Laboratory Science and Biotechnology, National Cheng Kung University Medical College, Tainan, Taiwan, ROC; ^5^ Center of Infectious Disease and Signaling Research, National Cheng Kung University Medical College, Tainan, Taiwan, ROC; ^6^ Advanced Optoelectronic Technology Center, National Cheng Kung University, Tainan, Taiwan, ROC; ^7^ Department of Neurochemistry, New York State Institute for Basic Research in Developmental Disabilities, Staten Island, New York, NY, USA; ^8^ Department of Neuroscience and Physiology, SUNY Upstate Medical University, Syracuse, NY, USA

**Keywords:** WWOX, TRAF2, p53, UV, cold shock

## Abstract

When COS7 fibroblasts and other cells were exposed to UVC irradiation and cold shock at 4°C for 5 min, rapid upregulation and nuclear accumulation of NOS2, p53, WWOX, and TRAF2 occurred in 10–30 min. By time-lapse microscopy, an enlarging gas bubble containing nitric oxide (NO) was formed in the nucleus in each cell that finally popped out to cause “bubbling death”. Bubbling occurred effectively at 4 and 22°C, whereas DNA fragmentation was markedly blocked at 4°C. When temperature was increased to 37°C, bubbling was retarded and DNA fragmentation occurred in 1 hr, suggesting that bubbling death is switched to apoptosis with increasing temperatures. Bubbling occurred prior to nuclear uptake of propidium iodide and DAPI stains. Arginine analog Nω-LAME inhibited NO synthase NOS2 and significantly suppressed the bubbling death. Unlike apoptosis, there were no caspase activation and flip-over of membrane phosphatidylserine (PS) during bubbling death. Bubbling death was significantly retarded in *Wwox* knockout MEF cells, as well as in cells overexpressing TRAF2 and dominant-negative p53. Together, UV/cold shock induces bubbling death at 4°C and the event is switched to apoptosis at 37°C. Presumably, proapoptotic WWOX and p53 block the protective TRAF2 to execute the bubbling death.

## INTRODUCTION

Frostbite is considered as traumatic injury and may lead to death [1–5]. Severe cold leads to hypothermia and damages in skin and other tissues or organs, which may result in limb amputations, organ damages, and death. Here, we propose that strategies can be developed to utilize the basic concept of frostbite, along with UV irradiation, to treat skin and other types of cancer.

Both extrinsic and intrinsic pathways are involved in the programmed cell death [6–8]. The extrinsic pathway is death receptor dependent. For example, stimulation of tumor necrosis factor (TNF) receptor by TNF-α results in activation of the downstream death pathway [9, 10]. In contrast, when cells possess damaged DNA or are subjected to apoptotic stress, the intrinsic pathway is activated. Cytochrome c release from the mitochondria into the cytosol occurs. Cytochrome c binds and causes the aggregation of the adaptor protein Apaf-1. Apaf-1 binds procaspase-9 molecules, which leads to assembly of pentameric Apaf-1/caspase-9. Further activation of downstream caspases 3 and 7 induces nuclear damage [11].

In this study we simulated frostbite *in vitro*. Cells were subjected to UV irradiation and then brief exposure to freezing conditions. The UV energy was at UVC range (wavelength at 265 nm). Gas formation was initiated in the nucleus. It appears that the UV energy was absorbed by the cells that allow relocation of many cytosolic proteins to the nucleus, which subsequently leads to generation of nitric oxide (NO)-containing gas in the nucleus. In most cases, the gas pushed out to form a short tunnel through the nuclear and the plasma membranes to form a single large bubble. In contrast, when cells were exposed to UV irradiation and then cultured at 37°C, the rate of bubble formation in these cells was significantly retarded, suggesting that functional proteins to block bubbling are at work at 37°C. We determined that tumor suppressor WW domain-containing oxidoreductase, designated human WWOX or FOR or mouse WOX1, participates in the UV irradiation-mediated apoptosis [12–15]. Here, we examined how WWOX/WOX1 acted in response to UV and cold shock, and determined whether components in the signal pathway, including NOS2, p53 and TRAF2 [7, 8], are involved in the bubbling death.

## RESULTS

### UV irradiation and cold shock induce “bubbling death” effectively at 4 and 22°C

**Figure 1:**
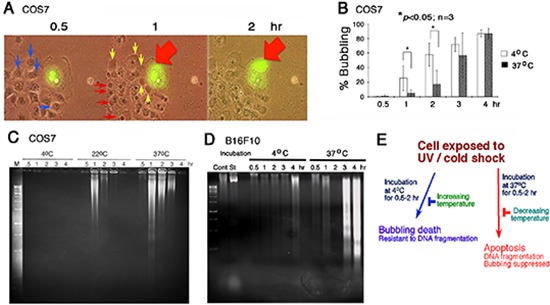
UV/cold shock induces nuclear membrane bubbling **(A)** COS7 cells, with or without expressing EGFP, were exposed to UV irradiation at 480 mJoule/cm^2^ and then incubated at 4°C for 5 minutes, prior to imaging by time-lapse microscopy at room temperature. Bubble formation (big red and small yellow arrows) from the nucleus is shown. Nuclear EGFP leaked into the gas bubble. See the enlarged pictures are at Supplementary Figure S1 for better resolution. **(B)** Under similar conditions, COS7 cells were exposed to UV irradiation (480 mJoule/cm^2^) and then cold shock (4°C for 5 min). The cells were then cultured at 4°C and 37°C, respectively, for indicated times. The extent of bubbling was counted. **(C, D)** Similarly, COS7 and B16F10 cells were exposed to UV (480 mJoule/cm^2^) /cold shock (4°C for 5 min) and then incubated at indicated temperatures for 0.5–4 hr. Internucleosomal DNA fragmentation was determined by agarose gel electrophoresis. **(E)** UV irradiation/cold shock-induced apoptosis is switched to bubbling cell death at low temperature, and vice versa.

When cells were subjected to UV irradiation and/or cold shock and then incubated at 4, 22 or 37°C for indicated durations, formation of a large bubble from the nucleus per cell was counted. UVC was used for all experiments. For example, when COS7 fibroblasts were overexpressed with EGFP (enhanced green fluorescent protein), followed by exposure to UV irradiation and then cold shock at 4°C for 5 min. Time-lapse microscopy was carried out at 22°C or room temperature for 2 hr. Gas was generated in the nucleus and pushed the nuclear and cytoplasmic membranes to pop out a big bubble (Figure 1A; Supplementary Figure S1 for enlarged pictures with better resolution). Nuclear EGFP relocated to the gas bubble with time of incubation. Each cell generated and released one bubble only (Supplementary Figures S1–S3). Neighboring cells, without expressing EGFP, also formed bubbles in a synchronized manner similar to that of the EGFP-expressing cells (Figure 1A). Indeed, upon exposure to UV, all cells underwent bubbling death in a synchronized manner (Supplementary Figure S3). Cold shock for 5 min at 4°C enhances the generation of bubbles. Herein, we designated the event as “bubbling death”.

UV alone induces bubbling of COS7 cells in a synchronized manner (Supplementary Figure S3), and cold shock could dramatically enhance the bubbling. For example, it took almost 1.5–2 hr for inducing 50% bubbling up to ~10 μm in diameter of the bubbles in UV only-treated cells, whereas it dropped down to 10–30 min when cells were exposed to UV/cold shock.

In similar experiments, COS7 cells were transiently overexpressed with ECFP or the WW domain of WWOX/WOX1 tagged with EGFP (EGFP-WWOXww), and subjected to UV irradiation/cold shock (4°C, 5 min). By time-lapse microscopy at room temperature, release of the ectopic ECFP or EGFP-WW domain from the nuclei to the bubble is shown (Supplementary Videos 1, 2). When cells were treated with UV/cold shock and then incubated at 4°C, 22°C, or 37°C for various durations, no major differences were observed regarding the morphological changes leading to bubbling death. When overexpressed, the ectopic WW domain protein localizes in the nucleus [15]. Also, generation of exosome-like particles of less than 1 μm in diameter [16], either intracellular or extracellular, is shown with time of bubbling (see the background in Supplementary Videos 1, 2).

### UV/cold shock-treated cells undergo bubbling death at low temperatures without DNA fragmentation

When COS7 cells were subjected to UV irradiation/cold shock and then cultured at 37°C, bubble formation was significantly retarded (Figure 1B). Similar results were observed by testing tumor necrosis factor (TNF)-sensitive L929 fibroblasts [15] (Supplementary Figure S4). That is, L929 cells were exposed to UV alone and then cold shock, followed by incubation at 4 and 37°C, respectively. In addition, when melanoma B16F10 cells were exposed to UV and then cold shock (4°C for 5 min), or cold shock first and then UV exposure. These cells were then cultured at 4, 22, or 37°C. Changes in the order of exposure to UV or cold shock did not alter the results of bubbling death at 4 or 22°C (data not shown). However, the bubbling event was totally blocked at 37°C (> 99%). We examined more than 10 cancer cell lines, including skin, breast and other cancer cells. In most cases, the bubbling death was retarded at 37°C (data not shown).

In stark contrast, we found that UV/cold shock-induced DNA fragmentation is blocked at low temperature but functions normally at 37°C. COS7 and B16F10 cells were exposed to UV/cold shock and then incubated at 4, 22, and 37°C for indicated times, followed by processing DNA fragmentation by agarose gel electrophoresis (Figure 1C, 1D). DNA fragmentation was blocked in COS7 cells at 4°C, but occurred at 22 and 37°C (Figure 1C). UV/cold shock-treated B16F10 cells were resistant to DNA fragmentation at 4°C, (Figure 1D) but exhibited bubble formation (data not shown). In contrast, DNA fragmentation was restored in UV/cold shock-treated B16F10 cells upon incubation at 37°C (Figure 1D), whereas bubbling was blocked totally at 37°C (Figure 1D). Taken together, UV/cold shock induces cells to undergo bubbling but resist DNA fragmentation at 4°C (Figure 1E). Upon increasing the temperatures to 37°C, bubbling death is retarded and DNA fragmentation occurs. The observations imply that switching from apoptosis to bubbling death is controlled by decreasing temperatures, and vice versa (Figure 1E).

### A nuclear gas bubble is generated per cell

**Figure 2:**
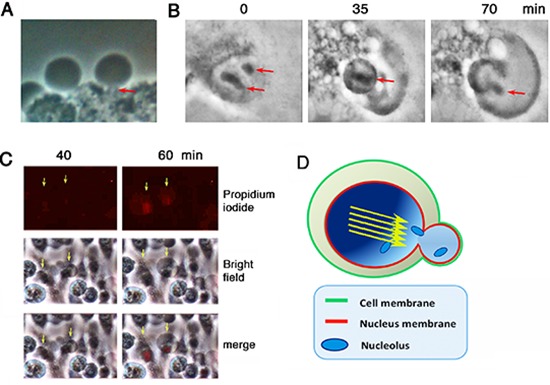
Bubbling from a gas jet stream COS7 cells were subjected to UV/cold shock and imaging by time-lapse microscopy at room temperature. **(A)** A representative COS7 cell exhibits a short gas tunnel or stalk (~1 μm in length; red arrow) connecting the cell and the bubble. **(B)** Release of nucleoli from a burst nucleus into the gas bubble is shown (red arrows). **(C)** Bubbling occurred prior to pickup of propidium iodide by the condensed nuclei. **(D)** A schematic model shows that the gas drives through the nucleus as a jet stream (yellow arrows) that punches out a single hole for generating a single bubble. The wall of the bubble is composed of the inner nuclear and outer cytoplasmic membranes.

It appears that under stimulation with UV/cold shock, the nuclear gas pushed forward as a jet stream in a single direction, so as to form a short stalk (~1–2 μm in length) to connect the bubble and the cell (see arrow; Figure 2A). Nucleoli were then released into the bubbles (see arrows; Figure 2B; Supplementary Video 3). Post bubbling for 30 min to 1 hr, cells were essentially dead as they failed to exclude trypan blue stain (> 95% cells) and their nuclei became positive for the propidium iodide stain (Figure 2C). A schematic graph shows a putative gas jet stream is formed and pops out to form a bubble. The wall of the bubble is probably enclosed by the outer plasma membrane and the inner nuclear membrane (Figure 2D).

**Figure 3:**
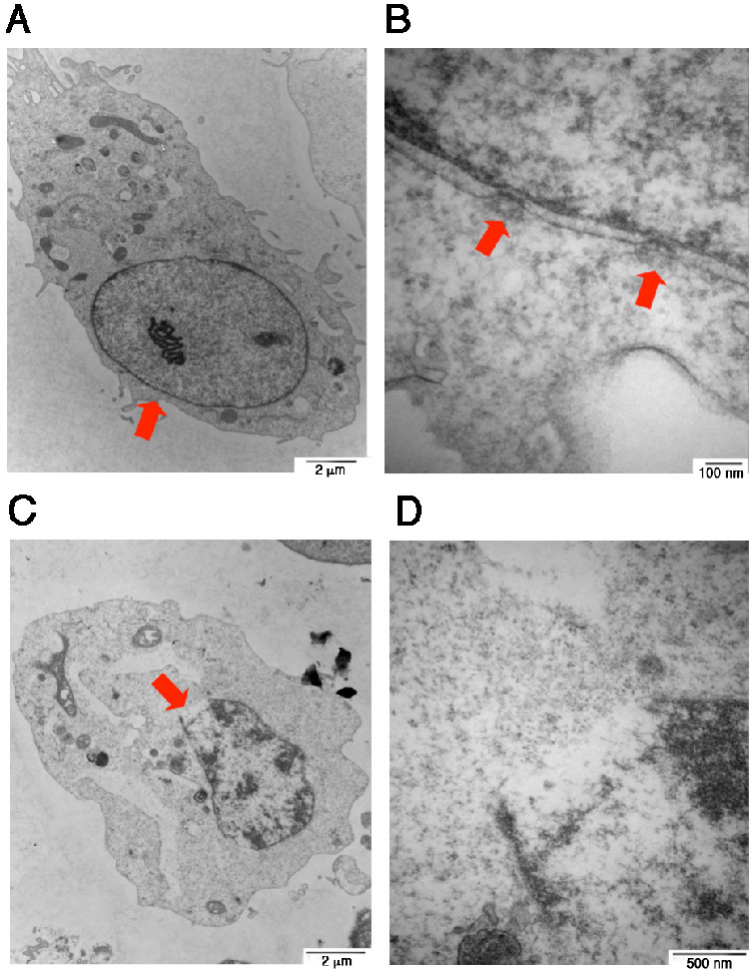
Electron microscopy of UV/cold shock-induced gas formation to cause damage to the nucleus COS7 cells were exposed to UV irradiation (480 mJoule/cm^2^) and then incubated at 4°C for 3 hours. Cells were fixed and prepared for electron microscopy analysis. **(A–B)** COS7 cells were without treatment. Intact nuclear pores are shown (see arrows). **(C–D)** A single gas hit to the nuclear wall is shown, which results in burst of the nucleus with a single opening (red arrow).

Finally, we attempted to verify the gas one-hit theory that a gas jet stream is formed in the nucleus and pops off to form one bubble per cell. COS7 cells were subjected to UV/cold shock, and then fixed for electron microscopy analysis [17]. In controls, COS7 cells received no UV/cold shock and their nuclei and nuclear pores were intact (see red arrows; Figure 3A–3B). When cells were subjected to UV/cold shock, a representative damaged nucleus underwent condensation and possessed an enlarged burst of the nuclear membrane (see the red arrow; Figure 3C–3D), suggesting that a gas jet stream hits the nuclear wall or pushes apart of a single nuclear pore that results in nuclear burst.

### Bubbling from nucleus is not identical to membrane blebbing

Bubbling from the nucleus cannot be considered the same as membrane blebbing. The membrane blebs are protrusions of the cell membrane, which exhibits many irregular bulges from the cell surface due to localized decoupling of the cytoskeleton from the plasma membrane [18, 19]. This event is reversible for the generation of small membrane blebs, whereas bubbling is irreversible for the formation of a final large bubble. We have previously demonstrated that U0126, an MEK1 inhibitor, prevents phorbol myristate acetate (PMA)-induced apoptosis of Jurkat T cells by causing membrane blebbing [20]. U0126 induces cells to undergo membrane blebbing to resist death [20].

### Bubbling death dose not exhibit flip-over of membrane phosphatidylserine (PS)

In contrast to apoptosis, there was no initial flip-over of phosphatidylserine (PS) toward the surface of cell membrane. For example, wild type *Wwox* MEF cells were exposed to UV/cold shock, and then incubated at 4°C for 30 min to 3 hr. No detectable positive signals were obtained using Annexin V stain (Supplementary Figures S5A). Similar results were observed using COS7 cells (data not shown). In positive controls, COS7 cells were transiently overexpressed with a Zfra expression construct by electroporation and then cultured for 24 hr. Flip-over of PS onto cell surface was shown as determined by Annexin V staining using flow cytometry (Supplementary Figure S5B). Zfra, known as zinc finger-like protein that regulates apoptosis, is a 31-amino-acid naturally occurring small protein [21–24]. We have shown previously that staurosporine- and Zfra-induced apoptosis are involved in flip-over of PS toward the surface of the cell membrane [21–24].

**Figure 4:**
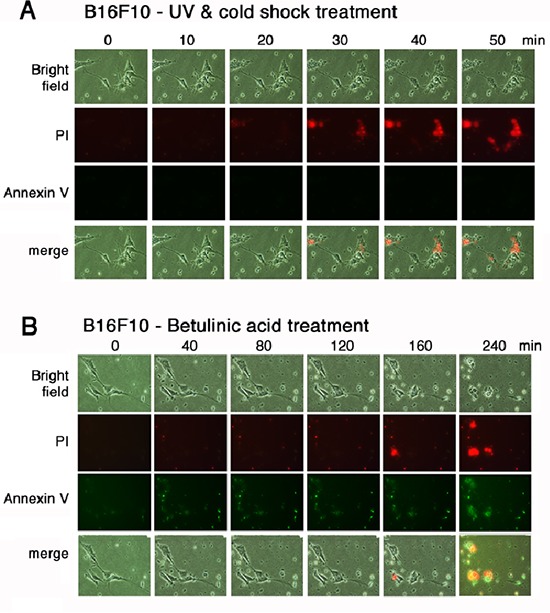
UV irradiation and cold shock did not cause flip-over of PS onto cell surface **(A)** B16F10 cells in culture were added aliquots of PI and Annexin V stains, exposed to UV irradiation (480 mJoule/cm^2^) and cold shock (4°C for 5 min), and subjected to time-lapse imaging at room temperature (2 min per frame). Little or no Annexin V-positive cells were observed. **(B)** Similarly, B16F10 cells were treated with betulinic acid (100 μM) and subjected to time-lapse imaging at room temperature (2 min per frame).

To further verify the aforementioned results, time-lapse microscopy was carried out using melanoma B16F10 cells. Aliquots of green fluorescent Annexin V and red fluorescent propidium iodide were added to the culture of B16F10 cells. The cells were subjected to UV/cold shock and time-lapse microscopy at room temperature. Again, damage to the nucleus that led to bubble formation occurred in less than one hour, as revealed by accumulation of the red fluorescent propidium iodide in the nucleus, whereas no green fluorescence showing the flip-over of PS to the cell surface was observed (Figure 4A). To induce mitochondrial apoptosis, B16F10 cells were treated with betulinic acid [20, 21]. Time-lapse microscopy showed the presence of green fluorescence prior to the cell death (Figure 4B).

### Bubbling death dose not cause damage to Golgi complex

COS7 cells were transiently overexpressed with ECFP specific for Golgi-targeting, followed by exposure to UV/cold shock. The ECFP protein was largely retained in the Golgi complex during bubbling death, as determined by time-lapse microscopy (Supplementary Videos 4A, 4B), suggesting that no damage to the Golgi complex. Again, exosome-like particles were released during bubbling death (Supplementary Videos 4A, 4B).

In comparison, COS7 cells were treated with staurosporine to induce apoptosis. A typical apoptotic cell death, including cell shrinkage and nuclear condensation, is shown, as determined by time-lapse microscopy (Supplementary Video 5).

### Starving cells prefer autophagic death but not bubbling death upon UV/cold shock exposure

When COS7 cells were pre-starved for 24–48 hr and then exposed to UV/cold shock, these cells exhibited increasing numbers of cytosolic autophagic vacuoles with time, along with whole cell shrinkage and nuclear burst (Supplementary Video 6). In most cases, the nuclei appeared to burst from inside out without generation of bubbles.

Also, COS7 cells were transiently overexpressed with LC3 tagged with EGFP, cultured for 24 hr, and then exposed to UV/cold shock. Time-lapse microscopy at room temperature revealed that during bubbling death, EGFP-LC3 was released to the generated bubble and no generated LC3 aggregates in the cytoplasm were shown (Supplementary Video 7), suggesting that no autophagy has occurred to induce LC3 degradation and aggregation.

### UV/cold shock induces the generation of nitric oxide (NO)-containing gas bubbles

**Figure 5:**
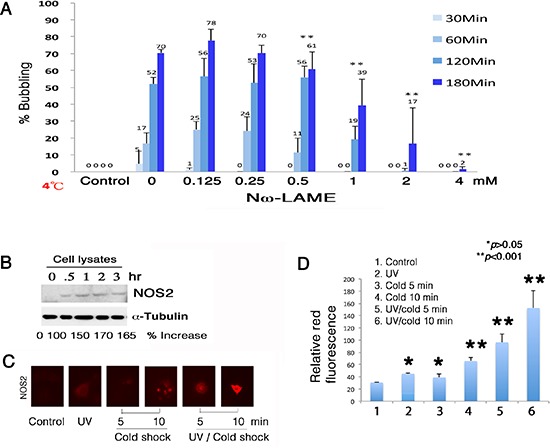
Suppression of bubbling by NOS2 inhibitor Nω-LAME **(A)** COS7 cells were pretreated with Nω-LAME (0.125–4 mM) for 30 min, followed by exposure to UV irradiation (480 mJ/cm^2^) and cold shock (4°C) for 30 to 180 min. **(B)** Induction of NOS2 expression by UV/cold shock was shown in 30 min. The levels of intracellular NFκB (p65) and α-tubulin remained largely unchanged. A representative data is shown from 2 experiments. **(C–D)** By immunofluorescence microscopy, UV/cold shock together significantly induced the expression of NOS2 in COS7 cells (*n* = 10; mean ± standard deviation; Student's *t*-test).

To determine the underlying mechanisms, Nω-nitro-L-arginine methyl ester hydrochloride (Nω-LAME), an analog of arginine, was shown to suppress the formation of gas bubbles (Figure 5A), suggesting that NO is in the generated gas. When COS7 cells were pretreated with Nω-LAME for 1 hr, and then exposed to UV and chilled at 4°C for indicated times, Nω-LAME blocked bubbling in a time- and a dose-dependent manner (Figure 5A). UV/cold shock rapidly induced the expression of nitric oxide synthase 2 (NOS2) in 30 min, as determined by Western blotting (Figure 5B). The cellular levels of α-tubulin were relatively unchanged (Figure 5B). Similarly, UV/cold shock significantly induced the generation of NOS2 in 5–10 min, as determined by immunofluorescence staining (Figure 5C and 5D). UV irradiation or cold shock alone was less effective (Figure 5C and 5D).

### WWOX/WOX1 and p53 are essential for UV/cold shock-induced nuclear damage and bubbling death

**Figure 6:**
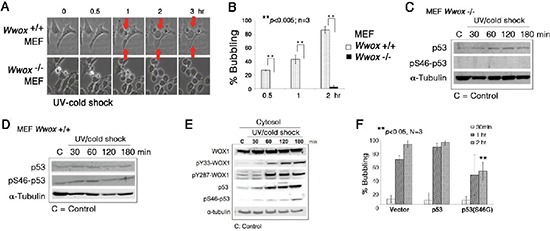
*Wwox* knockout MEF cells resist bubbling death **(A)** MEF *Wwox*^+/+^ and *Wwox*^−/−^ cells were exposed to UV irradiation (480 mJoule/cm^2^) and then cold shock at 4°C for 5 min, followed by chasing bubble formation at room temperature by time-lapse microscopy. **(B)** Similar experiments were carried out by treating MEF *Wwox*^+/+^ and *Wwox*^−/−^ cells with UV/cold shock, and incubated at room temperature for 0.5, 1 and 2 hr, respectively (~100 cells counted; *n* = 3; mean ± standard deviation; Student's *t*-test). **(C–D)** Again, *Wwox*^+/+^ and *Wwox*^−/−^ MEF cells were exposed to UV/cold shock, followed by incubation at 4°C for indicated durations. Expression of p53 and pS46–p53 is shown. α-tubulin is a loading control. A representative data is shown from 2 experiments. **(E)** In similar experiments, COS7 cells were subjected to UV/cold shock, and shown to have increased levels of cytosolic pY33-WOX1(WWOX), pY287-WOX1(WWOX), p53, and pS46–p53. **(F)** COS7 cells were transiently overexpressed with an empty vector, wild type p53, or dominant negative p53 (S46G–p53). These cells were cultured overnight and then exposed to UV/cold shock, followed by incubation at 4°C for indicated durations. The extent of bubbling death was counted.

We have previously demonstrated the essential role of tumor suppressors p53 and WWOX/WOX1 in causing UV irradiation-mediated apoptosis [12–14] and other events [21]. We established *Wwox* knockout mice and mouse embryonic fibroblasts (MEF) [25, 26]. When wild type *Wwox*^+/+^ MEF cells were subjected to UV/cold shock, these cells readily underwent bubbling death (Figure 6A and 6B; Supplementary Video 8). However, the knockout *Wwox*^−/−^ MEF cells resisted bubbling death by UV/cold shock (Figure 6A and 6B; Supplementary Video 9), suggesting the essential role of WWOX/WOX1 in the bubbling death.

Without WWOX/WOX1, p53 stability is dramatically reduced [13]. When wild type *Wwox*^+/+^ MEF cells were exposed to UV irradiation and cold shock at 4°C, these cells had an increased level of pS46–p53 by 15–50% (Figure 6C, 6D). However, pS46–p53 was not detectable in the knockout *Wwox*^−/−^ MEF cells treated with or without UV/cold shock (Figure 6C and 6D). Ser46 phosphorylation is essential for p53-mediated apoptosis [13]. In comparison, UV/cold shock-treated COS7 cells had increased levels of activated WWOX/WOX1 and p53 with phosphorylation at Tyr33 and Ser46, respectively (Figure 6E). Phosphorylation at Tyr287 in WWOX/WOX1 also occurred (Figure 6E). The observations indicate that protein phosphorylation can occur at 4°C. The Tyr287 phosphorylation is known to increase ubiquitination and degradation of WWOX/WOX1 [27]. Importantly, ectopic dominant-negative p53 (S46G–p53) effectively blocked the bubbling death in COS7 cells (Figure 6F), supporting an essential role of p53 in the bubbling death. Previously, we have demonstrated that p53 and WWOX/WOX1 act synergistically in inducing apoptosis under UV irradiation or environmental stress [12–14, 18].

### TRAF2 significantly retards bubbling death

**Figure 7:**
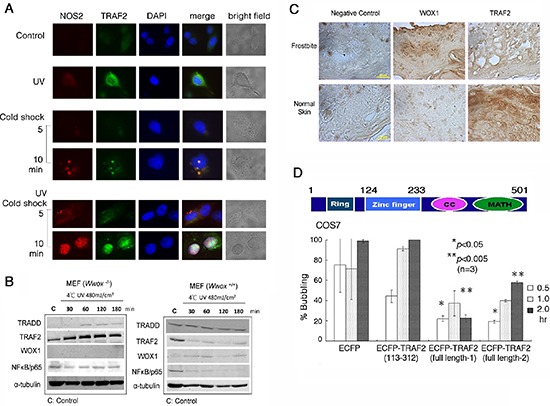
UV/cold shock induces NOS2 and TRAF2 expression **(A)** COS7 cell were exposed to UV irradiation (480 mJoule/cm^2^) and subsequent incubation at 4°C for 5 and 10 min. Cold shock alone weakly induced the expression of both endogenous NOS2 and TRAF2. Pre-exposure of cells to UV and then cold shock enhanced the generation of both proteins. UV alone rapidly induced the expression of TRAF2. **(B)** Down-regulation of antiapoptotic TRAF2 and NF-κB/p65 occurred when *Wwox* wild type MEF cells were exposed to UV (480 mJoule/cm^2^) and subsequent cold shock for 30 min to 3 hr. However, TRAF2 was upregulated in UV/cold shock-treated *Wwox* knockout MEF cells. **(C)** By immunohistochemistry, the expression of WWOX/WOX1 and TRAF2 in normal human skin and frostbitten skin is shown. Scale bars: 20 μm. **(D)** A schematic graph for the TRAF2 domains is shown. Ring = Ring-type zinc finger domain; CC = Coiled coil domain; MATH = MATH/TRAF domain. COS7 cells were transiently overexpressed with ECFP, truncated ECFP-TRAF2(113–312), and 2 identical full-length ECFP-TRAF2, respectively. UV (480 mJoule/cm^2^)/cold shock (4°C for 5 min)-induced bubbling death in cells expressing the indicated protein was counted (~100 cells counted; *n* = 3).

UVB activates TNF receptor-associated factor-2 (TRAF2), p53 and WWOX/WOX1 in the skin [12–14, 20, 28]. In COS7 cells, UV irradiation rapidly induced the expression of endogenous TRAF2 in the cytoplasm, and that subsequent cold shock caused upregulation and colocalization of TRAF2 and NOS2 in the nucleus in 10 min, as determined by immunofluorescence microscopy (Figure 7A). The nuclear accumulation of TRAF2 and NOS2 was greater than 95% (~100 cells counted; *n* = 3).

When *Wwox* wild type MEF cells were exposed to UV irradiation followed by cold shock for 30 min to 3 hr, both antiapoptotic TRAF2 and NF-κB/p65 were down-regulated, whereas the levels of proapoptotic TRADD were not reduced (Figure 7B). Under similar conditions, the level of TRAF2 was increased by 2–3 fold in *Wwox* knockout MEF cells (Figure 7B). The knockout cells were highly resistant to bubbling death by UV/cold shock (Figure 6A, 6B). By immunohistochemistry, normal human skin expressed a relative low level of WWOX/WOX1, but a high level of TRAF2 (Figure 7C). When human skin was subjected to frostbite, the WWOX/WOX1 level was dramatically increased (> 80% increase), and the TRAF2 level reduced by greater than 90% (Figure 7C).

To investigate the role of TRAF2 in regulating UV/cold shock, COS7 cells were transiently transfected with expression constructs of ECFP, truncated ECFP-TRAF2(113–312), and 2 identical full-length ECFP-TRAF2, respectively. The extent of protein expression was about 50–60%, as determined by fluorescence microscopy. When full-length TRAF2-expressing COS7 cells were subjected to UV/cold shock, bubbling death was significantly retarded, as compared to the ECFP control cells (Figure 7D). The *N*-terminal ring domain (amino acid #1–113) and *C*-terminal coiled coil and MATH domains (amino acid #313–501) were removed from the full-length TRAF2. The resulting ECFP-TRAF2(113–312) had a reduced suppression of bubbling death (Figure 7D). In these experiments, only cells expressing the indicated protein were counted.

## DISCUSSION

### Summary

We have discovered for the first time that UV irradiation/cold shock induces a novel type of programmed cell death, and this may provide a feasible approach for skin cancer treatment. Our key discovery is that UVC energy, coupled with low temperatures, mediates upregulation and accumulation of NOS2 in the nucleus, so as to generate a NO-containing gas per cell. The bubbling process does not require ATP (data not shown). The UV/cold shock-induced bubbling death functions effectively at low temperatures (4–22°C). Most strikingly, these cells resist DNA fragmentation at low temperatures, unless the temperatures go up to 37°C. At 37°C, bubbling death is dramatically suppressed in the UV/cold shock-pretreated cells. The observations imply that switching from apoptosis to bubbling death can be achieved by reducing temperatures, and vice versa.

We believe that NO, ROS, and other oxidizing gases may cause protein modifications. We found that cytosolic proteins undergo aggregation during bubbling death (data not shown). Also, there is a strong redox activity during the early stage of UV/cold shock exposure, prior to bubble formation. We have tried to isolate bubbles and determined the nature of proteins in the bubbles. This has been really difficult, as the bubbles are readily broken during sample preparation. Thus, the functional relevance of the nuclear proteins in the bubbles is unknown.

### Role of NO, NOS2, p53, WWOX and TRAF2

NO, NOS2, p53 and WWOX/WOX1 are crucial in the bubbling death, whereas the antiapoptotic TRAF2 blocks the cell death event. Suppression of NOS2 blocks the bubbling death. WWOX and p53 function in a synergistic manner in causing apoptosis under many types of stress conditions, including UV irradiation [12–15]. Both activated proteins physically interact using motifs surrounding the p-Tyr33 and p-Ser46 areas, and translocate to the mitochondria or nucleus to work synergistically in mediating apoptosis [12–15]. Whether WWOX and p53 bind and counteract the protective function of TRAF2 is unknown. In the absence of p53 and WWOX, the bubble formation is suppressed. The observations suggest that the nuclear accumulation of p53 and WWOX is needed to activate NOS2 to induce formation of NO.

In the frostbitten skin tissues, we showed that there is a greater than 90% downregulation of TRAF2, along with a dramatic upregulation of WWOX in humans. Unlike typical apoptosis, we did not observe PS flip-over onto the outer cell surface, mitochondrial apoptosis, and DNA fragmentation in the UV/cold shock-induced cell death at low temperatures. Unlike a recent report using ionizing irradiation [29], UV/cold shock-induced nuclear burst is not involved in activation of DNA damage-sensing proteins such as ATR, ATM, Chk1, and Chk2, as determined by Western blotting using specific antibodies (data not shown). Moreover, induction of DNA damage by an inducer, neocarzinostatin (10–40 μM) [29, 30], failed to cause bubbling death.

### Mechanistic insight

UV irradiation/cold shock rapidly causes upregulation and relocation of many cytosolic proteins to the nucleus. UV energy increases the expression of antiapoptotic TRAF2 and NF-κB/p65 and proapoptotic WWOX and p53, along with cytosolic NOS2 and membrane Hyal-2 and C1qBP (data not shown). Subsequent exposure of UV-treated cells to cold shock enhances accumulation of these proteins in the nucleus. NF-κB/p65 is a downstream effector of the TNF signaling, which blocks TNF-mediated cell death and thereby provides cancer survival [31, 32]. Hyal-2 acts as an alternative receptor for TGF-β1 and a binding protein for WWOX [17]. Serum complement C1q participates in cancer suppression via interaction with C1qBP and downstream WWOX [33]. WWOX plays a role in the hypoxic response [34]. Whether HIF1α is involved in the UV/cold shock response is unknown.

### NOS2 and TRAF2

NOS2 is one of the nitric oxide synthase family proteins [35–37], and is responsible for the generation of NO-containing gas in the nucleus. Functional nitric oxide synthase has been shown in the nucleus [37]. In response to UV/cold shock, NOS2 becomes colocalized with TRAF2. Whether NOS2 and TRAF2 interact with each other and co-translocate to the nucleus remains to be established. Also, whether NOS2 binds p53 and WWOX is unknown. NOS2-regulated NO formation needs further conformation by using other specific methods. Similarly, how the jet stream pushes out in a directional manner through the nuclear and cell membranes is unknown and remains to be established.

### Cold shock and protein relocation

UV alone is able to upregulate WWOX/WOX1, p53, NF-κ/p65, and TRAF2, plus C1qBP and Hyal-2 (data not shown). In contrast, cold shock alone is sufficient to induce the expression of NOS2, indicating the synthesis of mRNA and proteins at low temperature in mammalian cells. In combination, UV irradiation with subsequent cold shock promotes the accumulation of the aforementioned proteins in the nucleus. How the cold shock drives proteins to relocate to the nucleus is very intriguing. Yet, UV/cold shock induces NO-containing gas generation and bubble formation in an ATP-independent manner, which leads to cell death *in vitro* and may provide explanation to our understanding of the clinical feature of frostbite in humans.

### Translocation of nucleolar proteins and exosome release

As determined by time-lapse microscopy, a majority of the nuclear and nucleolar proteins go to the bubble, and a minor portion may leak to the cytoplasm. We have tried to isolate bubbles and determined the nature of proteins in the bubbles. This has been really difficult, as the bubbles go readily broken during sample preparation. The functional relevance of the nuclear proteins in the bubbles is unknown. Furthermore, where there is a connection between the nuclear events of bubbling to the cytosolic membrane event of exosome release has yet to be established. Generation of exosomes could occur as the side effect (or counteractive forces) generated by the gas jet stream.

### Inhibition of bubbling death by TRAF2

TNF-induced apoptosis can be turned off by activating NF-κB through TRAF2 [28]. TRAF2 has an *N*-terminal RING-type zinc finger domain, followed by a zinc finger domain, a coiled coil domain, and a MATH/TRAF domain at the *C*-terminus. Deletion of the *N*-terminal RING-type zinc finger domain of TRAF2 resulted in partial loss of its activity in blocking UV/cold shock-induced bubbling. Further analysis showed that the central zinc finger domain confers resistance to the effect of UV/cold shock. It has been reported that TRADD directly interacts with TRAF2, and then activates NF-κB for inducing the antiapoptotic event [8, 38–40]. In UV-activated cell apoptosis, TRAF2 promotes cell survival via activating NF-κB and inhibiting p53 from binding to the mitochondria and blocking the release of cytochrome C [7, 28, 41].

### Conclusion

UV/cold shock induces bubbling death of cells at low temperatures (4–22°C). The event is p53- and WWOX-dependent. There is little or no chromosomal DNA fragmentation at low temperatures, indicating that apoptotic death is blocked. Upon raising the temperature up to 37°C, bubbling death is dramatically retarded in many types of cells, whereas DNA fragmentation occurs. That is, switching from bubbling death to apoptosis can be achieved by raising temperatures. Overall, we have discovered a novel bubbling death operating at low temperatures, and that apoptosis is blocked.

## MATERIALS AND METHODS

### Cell lines, transient gene expression in cells, and stable gene transfectants

Monkey kidney COS7 fibroblasts, murine L929 fibroblasts, murine melanoma B16F10, human squamous cell carcinoma SCC9 and SCC15, human colon HCT116, MEF *Wwox*^−/−^ and *Wwox*^+/+^ cells, and 10 other types of cancer cell lines were cultured and used for experiments [12, 13, 15, 25]. COS7 and L929 were mainly used for experiments. Where indicated, cells were transfected by electroporation (BTX ECM 630 electroporator, Genetronics) with a specific mammalian expression construct for transient expression [12, 13, 15]. Or, the cells were treated with G418 for establishing stable gene transfecctants [12, 13, 15].

### cDNA expression constructs

A variety of WWOX/WOX1 and p53 cDNA expression constructs, in pEGFP-C1, pEYFP-C1, pECFP-C1, and pDsRed monomer, were made as described [12, 13, 15, 19]. The following primers were used to construct TRAF2 cDNAs: 1) TRAF2 (full-length)-pEGFP-C1, forward: CAAGCTTGCATGG CTGCAGCCAGTGTGACTTC CCCTGGCTCCC; reverse: GCGCGAA GCTTCTAGAGTCCTGTTAGGT CCACAATAGCTTTGATG. 2) TRAF2(124–233)-pEGFP-C1, forward: ATTGAATTCTATGTGCCACGA AGGAC; reverse: AATGAATTCTTACT GCAGGTTCT CAGTCTCC.

### Chemicals and antibodies

Polyclonal antibodies against WWOX, p53, pS46–p53, TRADD, FADD, TRAF2, ERK, and pERK, and monoclonal antibodies against NOS2, C1qBP, NF-κB/p65 and p53 were purchased from Santa Cruz Biotechnology. Lamin A/C polyclonal antibody and an antibody kit against proteins in sensing DNA damage (e.g. ATR, ATM, Chk1, Chk2, and others) were from Cell Signaling. Homemade polyclonal antibodies against WWOX/WOX1 and pTyr33-WOX1 were generated in rabbits as described [12, 13, 15]. Phospho-Ser14 WWOX/WOX1 polyclonal antibodies were made in rabbits against a synthetic peptide CAGLDDTD-pS-EDELPPG (amino acid sequence #7–21; Genemed Synthesis) [12, 13, 15]. The cysteine residue was added to the *N*-terminus to facilitate conjugation of the peptide to Keyhole Limpet Hemocyanin (KLH). Similarly, polyclonal antibodies against the phospho-Tyr287 WOX1 antibody were also made (amino acid sequence #286 to 299) using a synthetic peptide D-pY-WAMLAYNRSKLC. Two corresponding peptides WOX1(7–21) and WOX1(285–299) without phosphorylation were also made, respectively, and used for antibody production. An approved protocol for animal use was obtained from the Institutional Animal Care and Use Committee of the National Cheng Kung University Medical College. Nω-LAME, DAPI, and staurosporine were from Calbiochem.

### Immunofluorescence microscopy, electron microscopy, and time-lapse microscopy

COS7 cells were irradiated with UV 480 mJ/cm^2^ and then subjected to cold shock for 30, 60, 120, 180 minutes at 4°C, respectively. Where indicated, the cells were incubated at room temperature or 37°C. In controls, cells were not treated or exposed to UV or cold shock only. Immunostaining was carried out using specific antibodies against TRAF2 and WWOX/WOX1, or other combinations. Electron microscopy was carried out as described [17]. Time-lapse microscopy was also carried out for UV/cold shock-treated COS7 and other cells. Confocal microscopy was then carried out using Nikon C1-Si Laser Scanning Confocal Microscope [17].

### Immunohistochemistry (IHC), co-immunoprecipitation and Western blotting

IHC, co-immunoprecipitation and Western blotting were carried out, as described [12–15, 17]. Antibody against WWOX/WOX1 was used for co-immunoprecipitation [12–15, 17, 22]. For IHC, skin tissue sections from patient were immersed in blocking solution (2% BSA) for 1 hr, followed by adding an aliquot of antibody against WWOX/WOX1 or TRAF2 for 2 hr incubation at room temperature. Following washing with PBS for 3 times, the tissue sections were incubated with aliquots of horseradish peroxidase (HRP)-conjugated secondary antibodies, and the resulting color was then developed using an immunohistochemical staining kit (Dako). In controls, no primary antibodies were used. The images were analyzed by an NIH Image J program.

## SUPPLEMENTARY FIGURES



## Abbreviations

EGFP: enhanced green fluorescent protein
PS: phosphatidylserine
TRAF2: TNF receptor-associated factor 2
TNF: tumor necrosis factor
WWOX (WOX1, FOR): WW domain-containing oxidoreductase
NOS2: nitric oxide synthase 2
NO: nitric oxide
Nω-LAME: Nω-nitro-L-arginine methyl ester hydrochloride
