# Supplementary material for: UV irradiation/cold shock-mediated apoptosis is switched to bubbling cell death at low temperatures
Source: Oncotarget. 2015 Feb 10;6(10):8007–18. doi: 10.18632/oncotarget.3153 (PMC4480731; doi:10.18632/oncotarget.3153)
Supplement: Supplementary file 1 [file oncotarget-06-8007-s001.pdf]

## SUPPLEMENTARY FIGURES

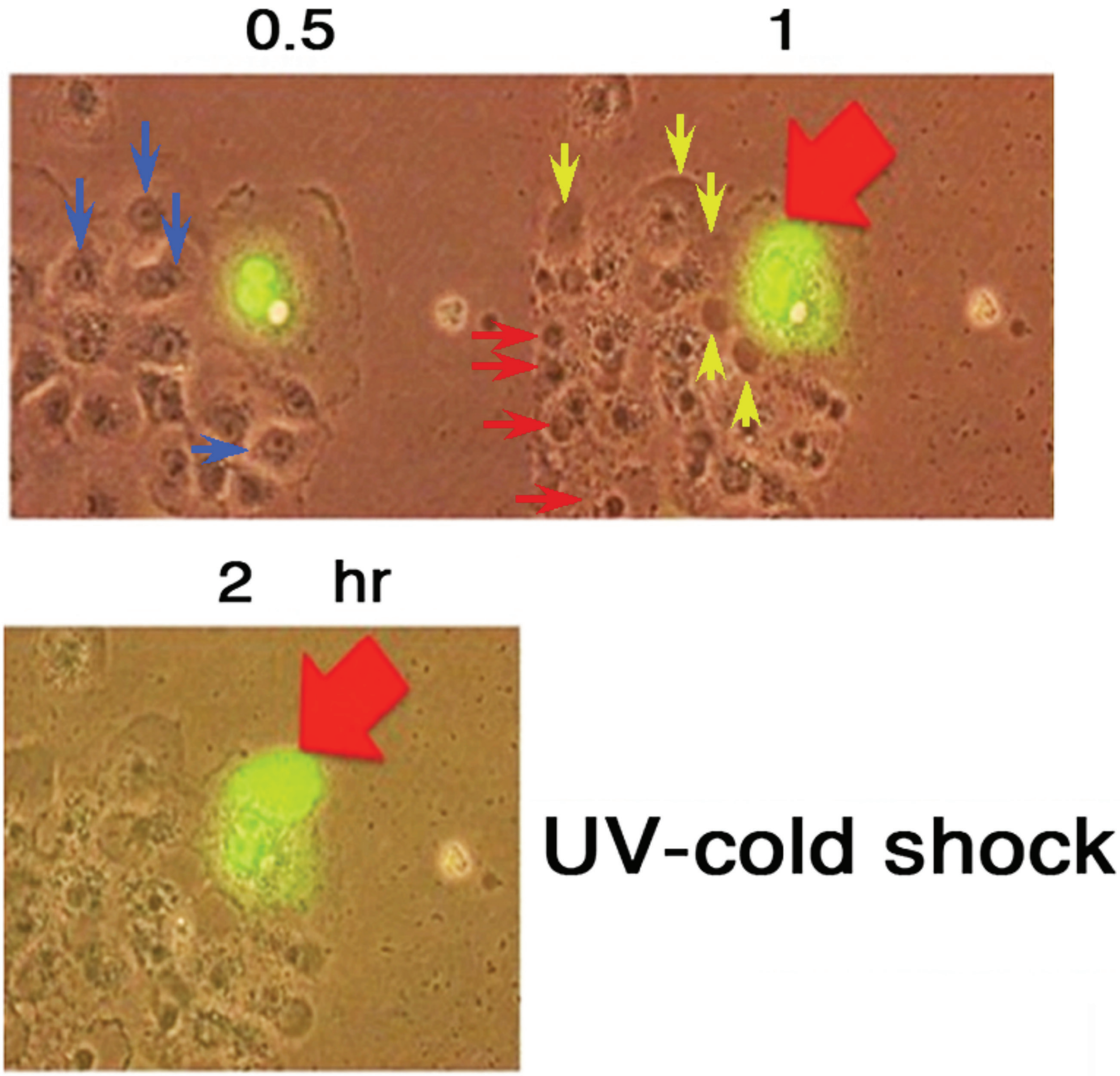


**Supplementary Figure S1: UV irradiation and cold shock induce nuclear gas formation and bubbling death.** EGFP-expressing COS7 and neighboring control cells were exposed to UV irradiation at 480 mJoule/cm$^2$ and then incubated at 4°C for 5 minutes. Time-lapse microscopy was then carried out at room temperature. Bubble formation (big red arrows) from the nucleus is shown. Nuclear EGFP leaked into the gas bubble. Post-treatment from 30 min (blue arrows) to 60 min (small red arrows), nuclear condensation occurred. Gas bubble formation is shown from the first hour (yellow arrows) and the second hour. Enlarged pictures are from Figure 1A.

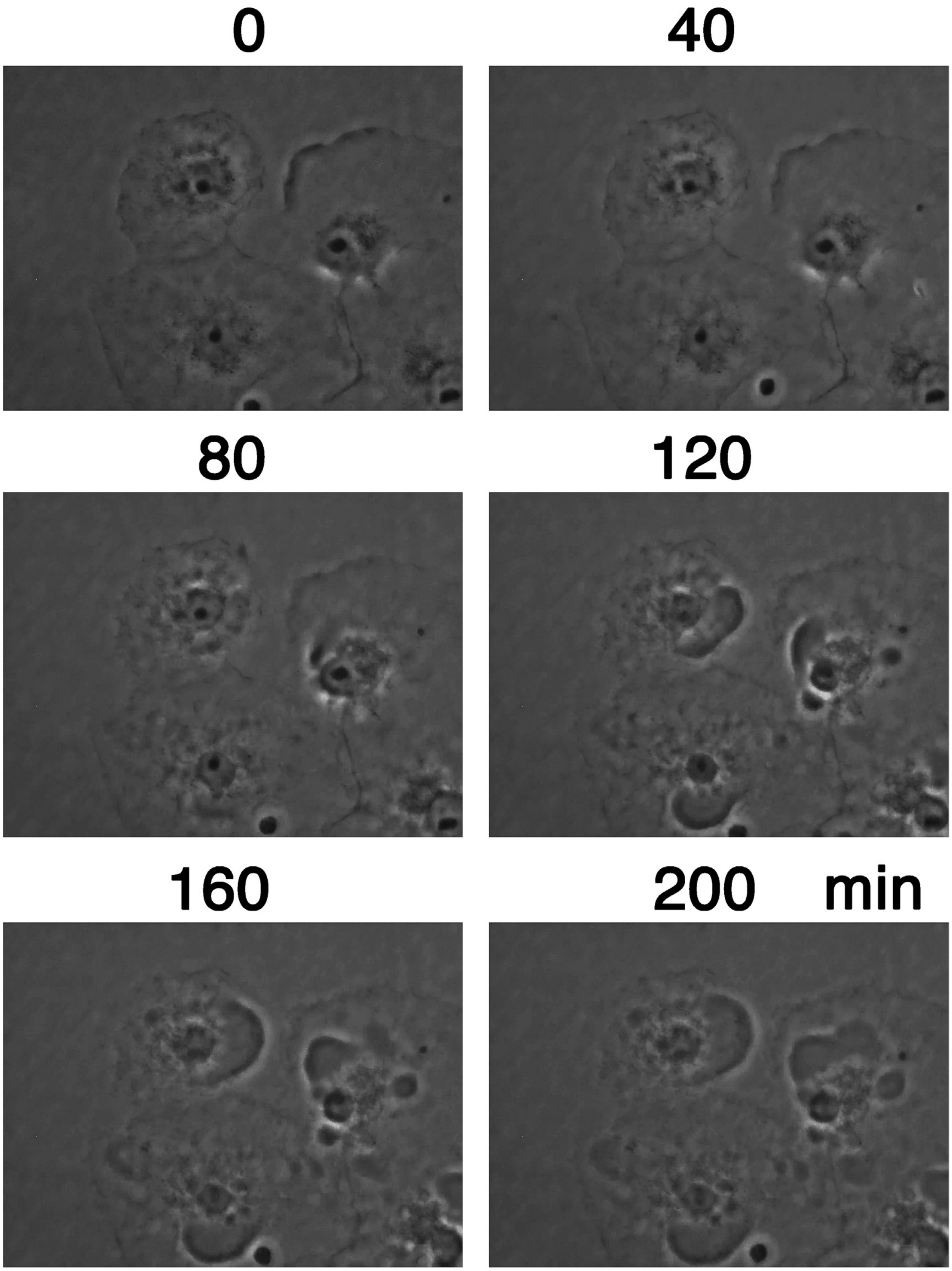


**Supplementary Figure S2: Formation of a nuclear gas bubble in a COS7 cell upon exposure to UV/cold shock.** Generation of a gas bubble from the nucleus is shown in a representative COS7 cell upon exposure to UV irradiation (480 mJoule/cm$^2$) and then cold shock (4°C for 5 min). Time-lapse microscopy was carried out at room temperature or 22°C.

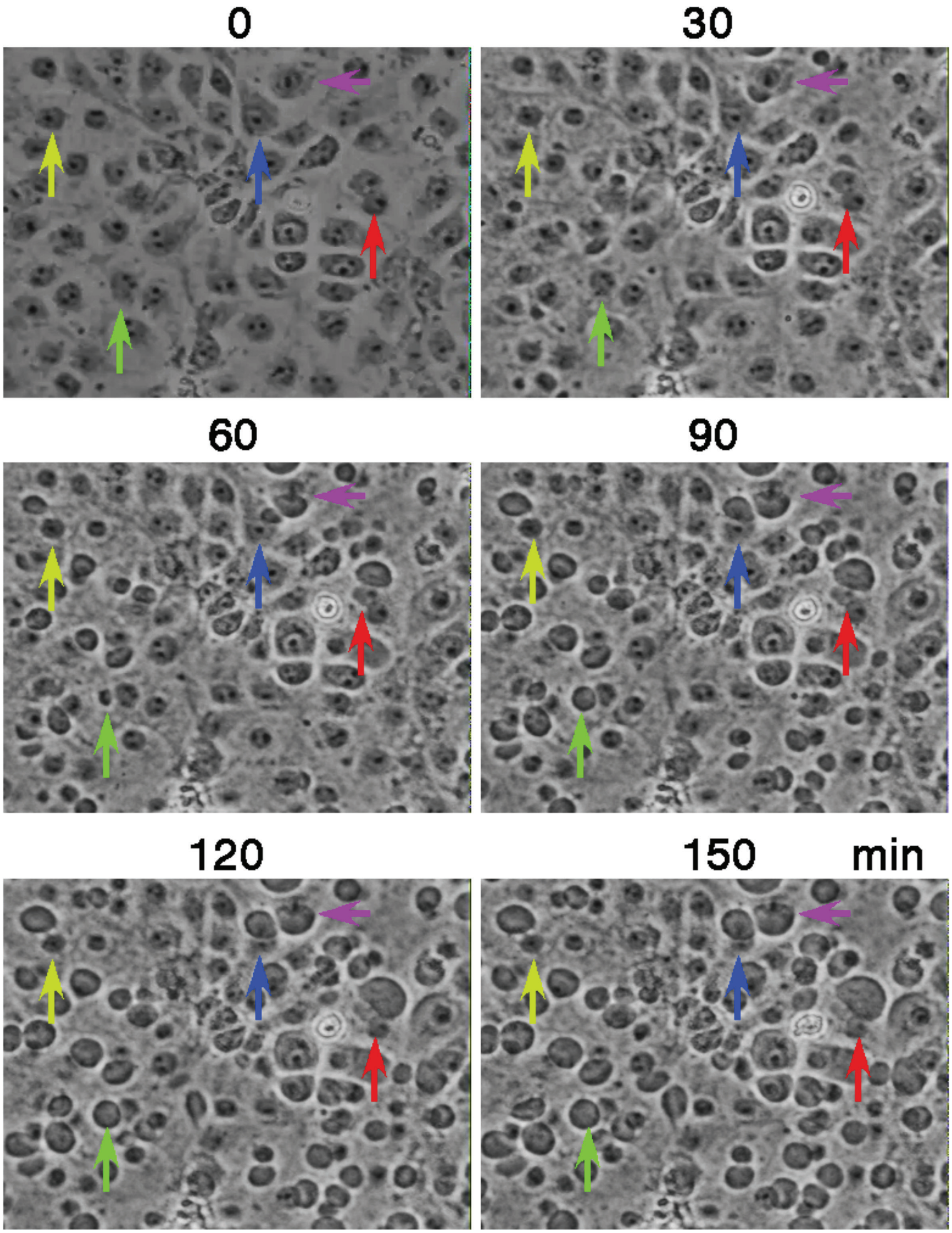


**Supplementary Figure S3: COS7 in bubbling death.** COS7 cells were subjected to UV irradiation. UV-induced bubbling death at room temperature was determined by time-lapse microscopy (5 min per frame). Five randomly selected cells were shown (see arrows).

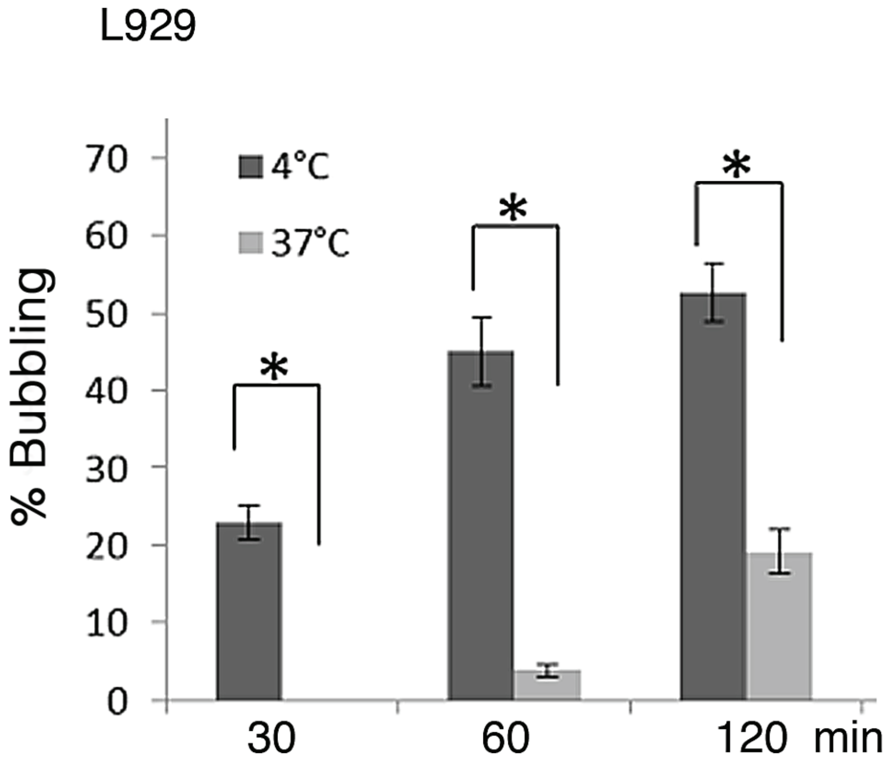


**Supplementary Figure S4: Mouse L929 cells resist UV/cold shock-induced bubbling death at 37°C.** Mouse L929 cells were exposed to UV alone (480 mJoule/cm$^2$) and then cold shock at 4°C for 5 min, followed by incubation, respectively, at 4 and 37°C. The extent of bubbling was measured.

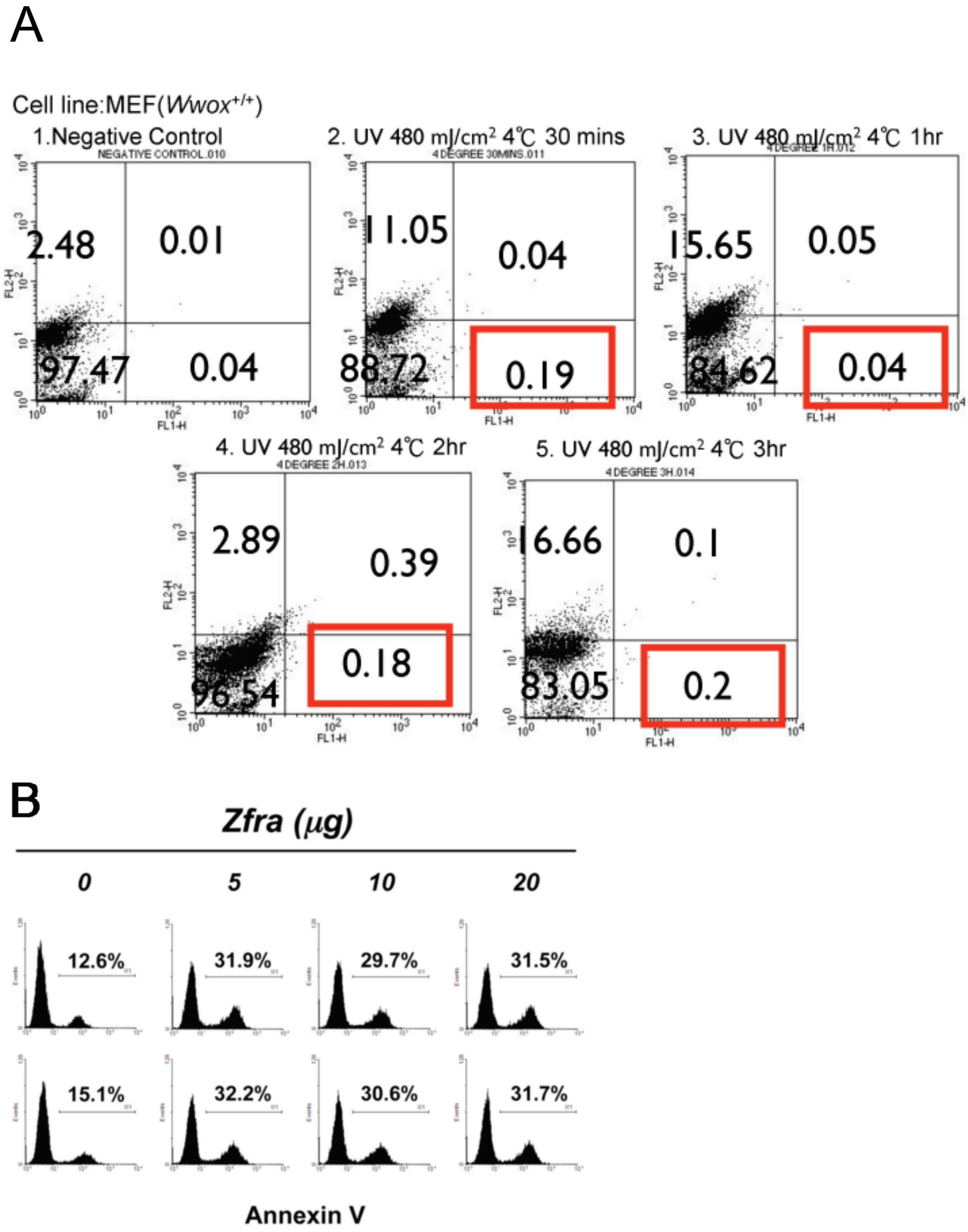


**Supplementary Figure S5: UV irradiation and cold shock did not induce flip-over of phosphatidylserine (PS) onto cell surface.** **(A)** Wild type Wwox MEF cells were exposed to UV irradiation (480 mJoule/cm$^2$) and then cold shock at 4°C for indicated durations. By flow cytometry, little or no Annexin V-positive cells were shown (see red boxes). **(B)** In positive controls, COS7 cells were electroporated with various amounts of Zfra constructs (Zfra-pCR3.1) by electroporation and then cultured for 24 hr. Flow cytometry revealed flip-over of PS onto cell surface, as determined by Annexin V staining (in duplicates). Overexpressed Zfra is known to induce apoptosis [21–24].

## SUPPLEMENTARY VIDEOS

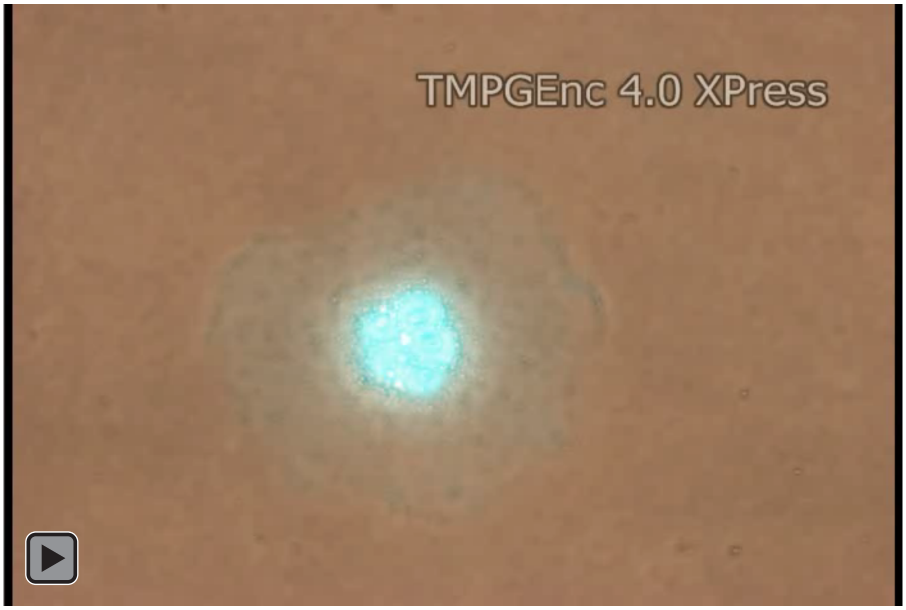


**Supplementary Video 1: UV/cold shock induces nuclear gas generation and bubble formation.** COS7 cells were transiently overexpressed with ECFP. These cells were exposed to UV irradiation (480 mJoule/$cm^2$) and subsequently cold shock at 4°C for 5 min. Release of ECFP from the nucleus to the generated bubble is shown. Time-lapse microscopy was carried out at room temperature for 2 hr.

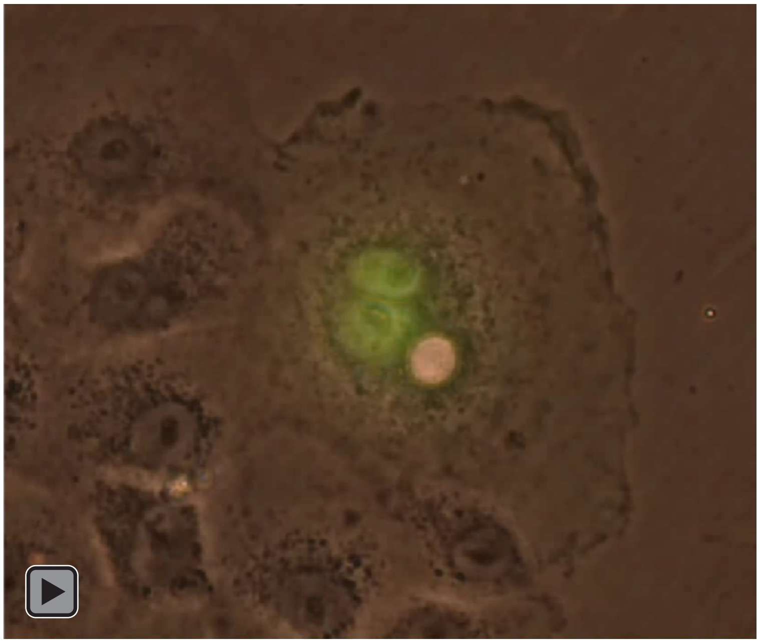

**Supplementary Video 2: UV/cold shock induces leaking of EGFP-WW protein into the generated membrane bubble.** COS7 cells were transiently overexpressed with nuclear targeting EGFP-WW domain [15], and then exposed to UV irradiation (480 mJoule/cm$^2$) and subsequently cold shock at 4°C for 5 min. The cells were imaged by time-lapse microscopy at room temperature for 2 hr. Exosome-like particles (~500 nm in diameter) were released by this and surrounding cells with time.

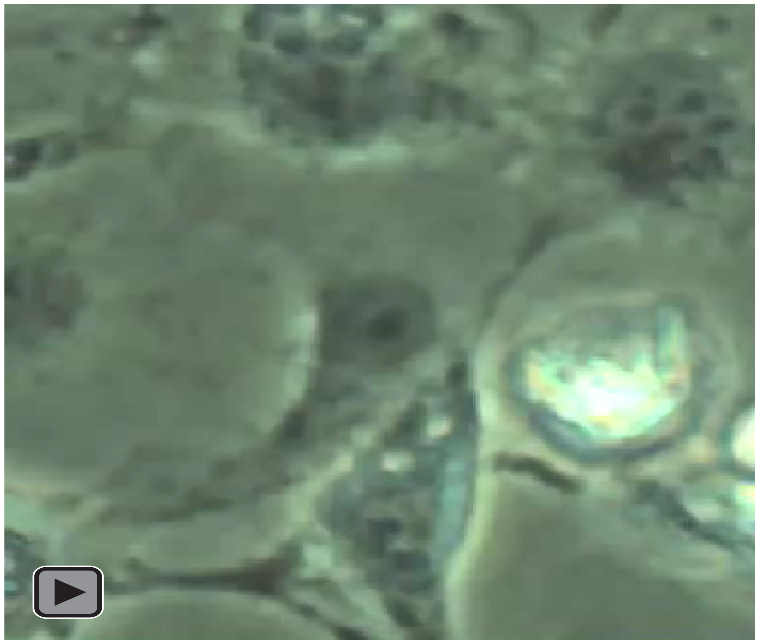

**Supplementary Video 3: Release of nucleoli into the generated nuclear bubble.** COS7 cells were exposed to UV irradiation (480 mJoule/cm$^2$) and subsequently cold shock at 4°C for 5 min. The cells were imaged by time-lapse microscopy at room temperature for 2 hr. The nuclei popped open, followed by release of nucleoli into the generated nuclear bubbles. See the image in the center of the field.

(A)

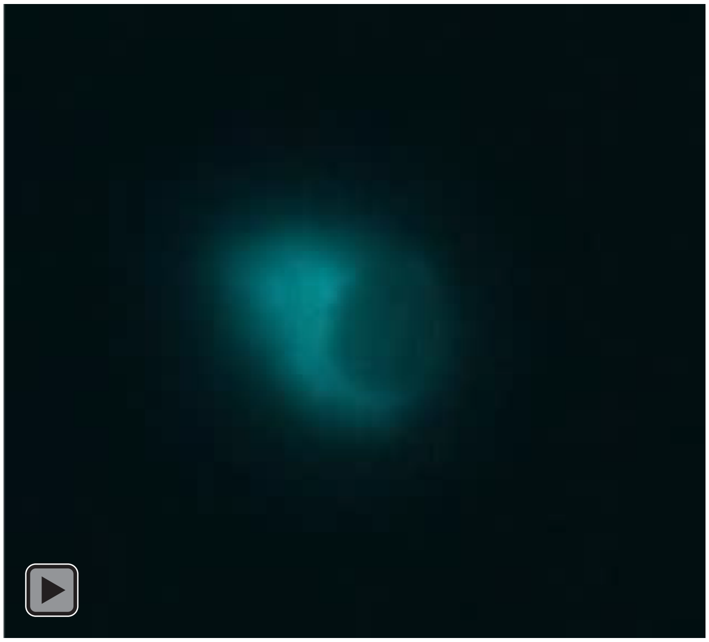

(B)

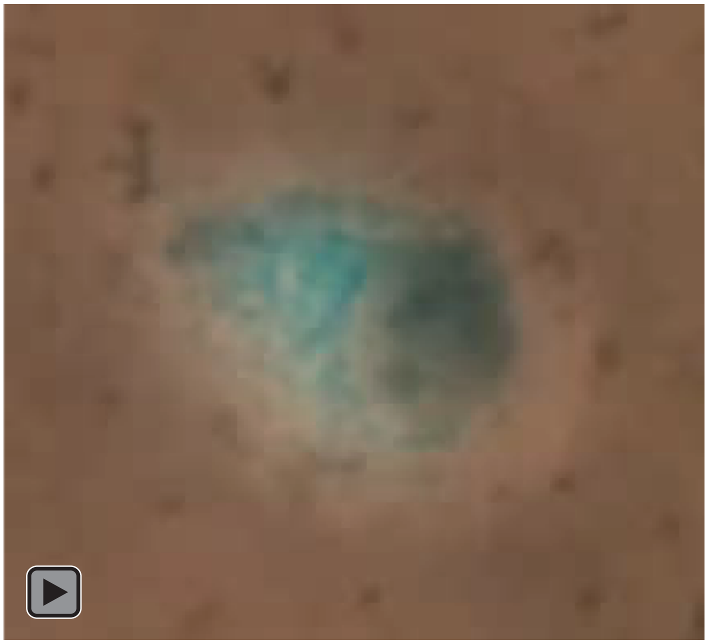

**Supplementary Video 4: Little or no release of ECFP protein in the Golgi complex during bubbling. (A)** COS7 cells were transiently overexpressed with Golgi-targeting ECFP. Post culture for 24 hr, these cells were exposed to UV irradiation (480 mJoule/$cm^2$) and cold shock at 4°C for 5 min, and then imaged by time-lapse microscopy at room temperature for 2 hr. The ECFP protein was largely retained in the Golgi complex during bubbling. **(B)** A video shows the imaging merged from both bright field and ECFP.

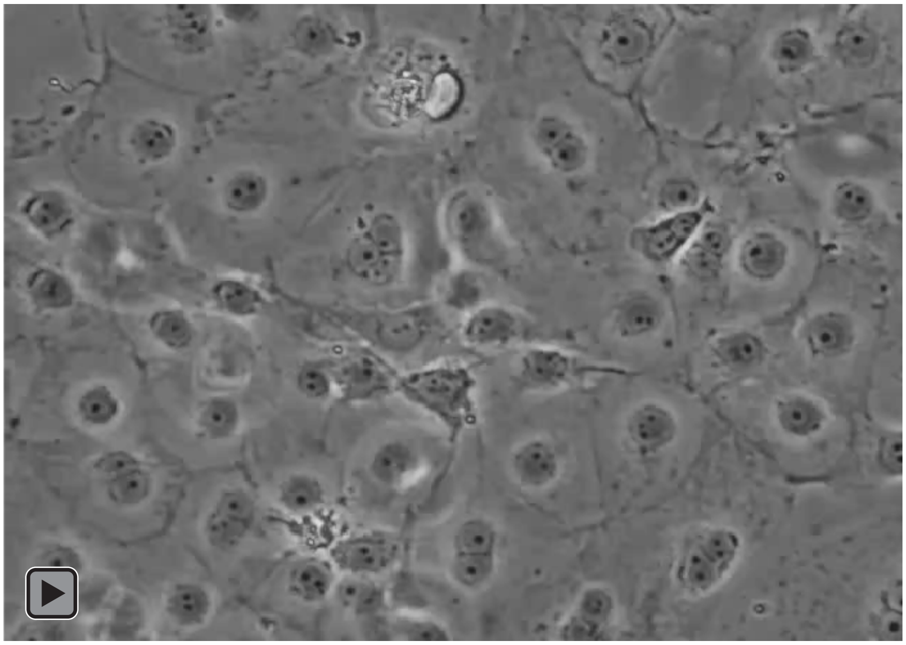

**Supplementary Video 5: Staurosporine induces apoptosis in COS7 cells.** COS7 cells were treated with staurosporine (1 µM). The cells were imaged by time-lapse microscopy for 4 hr at room temperature (2 min per frame). Similar results were shown at 37°C imaging.

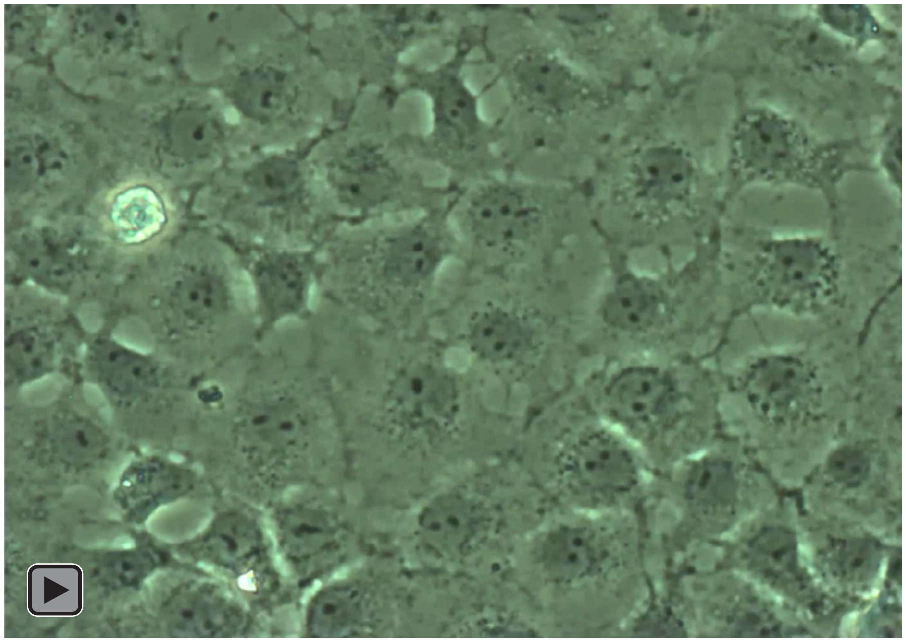

**Supplementary Video 6: Starvation induces autophagic death and blocks bubbling in COS7 cells.** COS7 cells were starved for 24 hr under serum-free culture conditions, followed by exposure to UV irradiation (480 mJoule/$cm^2$) and cold shock (4°C for 5 min). The cells were imaged by time-lapse microscopy at room temperature (2 min per frame). A representative data from 3 experiments is shown.

**(A)**

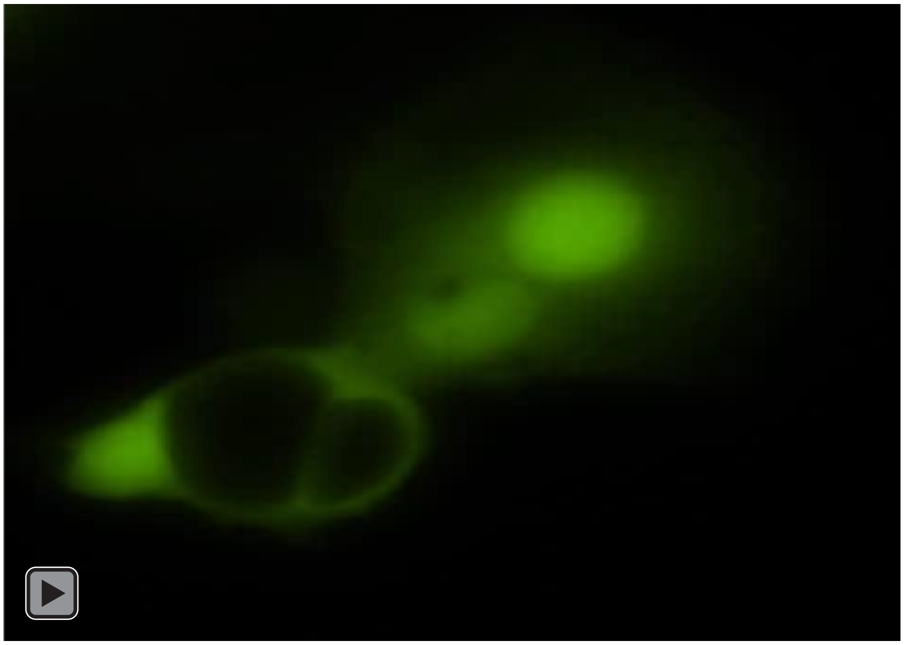

**(B)**

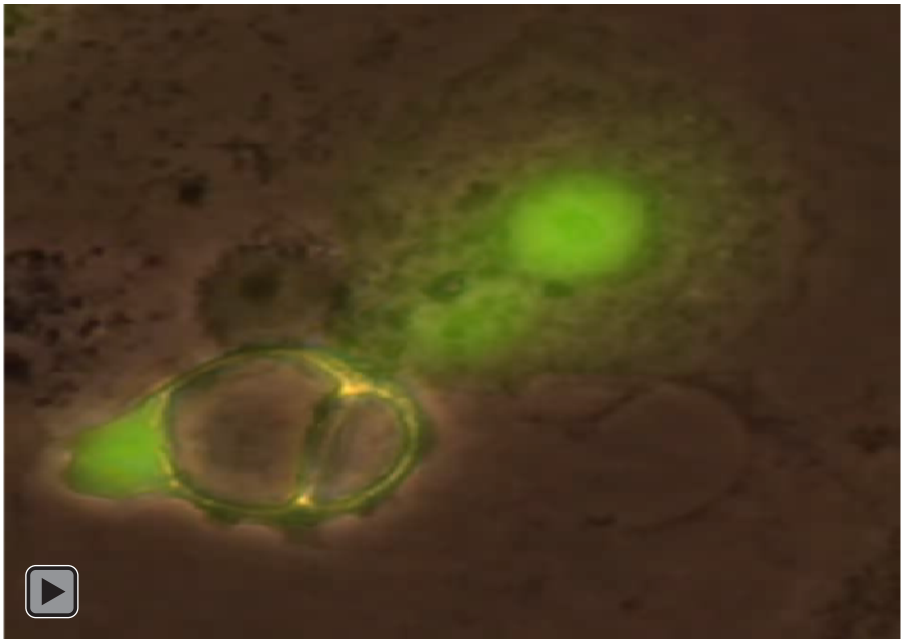

**Supplementary Video 7: No autophagic activation during bubbling. (A)** COS7 cells were transiently overexpressed with LC3 tagged with EGFP. Post culture for 24 hr, these cells were exposed to UV irradiation (480 mJoule/$cm^2$) and cold shock at 4°C for 5 min, and then imaged by time-lapse microscopy at room temperature for 2 hr. No LC3 punctates were observed in the cytoplasm. **(B)** A video shows the imaging merged from both bright field and EGFP (2 min per frame).

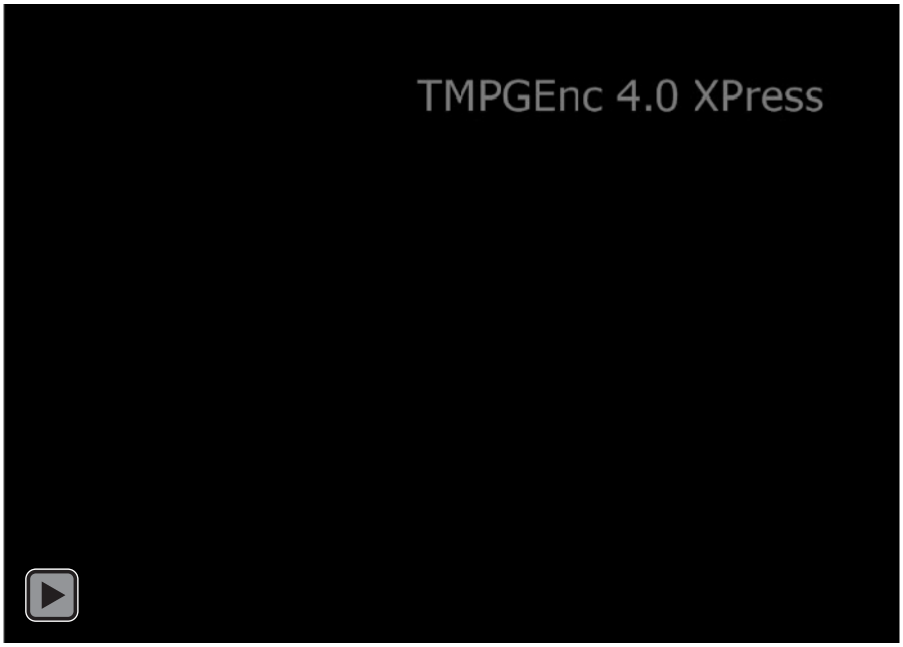


**Supplementary Video 8: UV/cold shock induces bubble formation in the wild type *Wwox*$^{+/+}$ MEF.** Wild type *Wwox*$^{+/+}$ MEF cells were subjected to UV irradiation (480 mJoule/cm$^2$) and cold shock at 4°C for 5 min, and then imaged by time-lapse microscopy at room temperature for 2 hr. One bubble per cell was generated.

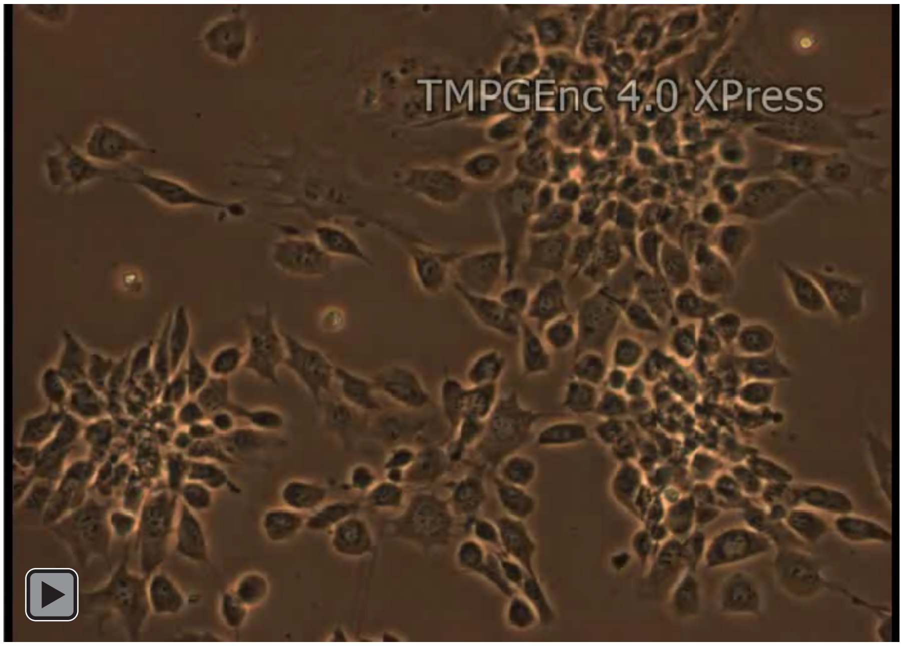


**Supplementary Video 9: UV/cold shock does not effectively induce cell membrane bubble formation in the knockout *Wwox*$^{-/-}$ MEF.** Knockout *Wwox*$^{-/-}$ MEF cells were subjected to UV irradiation (480 mJoule/cm$^2$) and cold shock at 4°C for 5 min, and then imaged by time-lapse microscopy at room temperature for 2 hr.
